# *meso*-Dimethylaminonaphthyl-BODIPY Derivatives as Bioimaging Probes to Monitor Intracellular pH and Label Lysosomes and Lipid Droplets

**DOI:** 10.1021/acs.jpcb.5c00926

**Published:** 2025-07-01

**Authors:** Raquel C. R. Gonçalves, Sónia C. S. Pinto, Filipe Teixeira, Efres Belmonte-Reche, Milene Costa da Silva, Juan Gallo, Susana P. G. Costa, M. Manuela M. Raposo

**Affiliations:** 1 Chemistry Centre of the University of Minho, Campus of Gualtar, Braga 4710-057, Portugal; 2 Advanced (Magnetic) Theranostic Nanostructures Lab, 246702 International Iberian Nanotechnology Laboratory, Av. Mestre José Veiga s/n, Braga 4715-330, Portugal

## Abstract

Fluorescence-based probes are a powerful tool for monitoring living systems in real time with spatiotemporal precision. 3-Difluoroborodipyrromethene (BODIPY) derivatives are widely used as bioimaging probes due to their excellent photophysical properties, high quantum yields, and chemical stability. In this context, we report two BODIPY-based fluorescent probes for visualizing lysosomes and lipid droplets as well as tracking intracellular pH variations within living cells. Computational studies, employing molecular dynamics (MD) simulations and the simplified Tamm–Dancoff approximation (sTDA), were conducted to predict the absorption properties of these derivatives in different chemical environments. These studies accurately reproduced the experimental absorption bands and revealed solvent-dependent spectral shifts, providing insights into the photophysical behavior of the compounds. *In vitro* studies using HeLa cells demonstrated that the BODIPY derivatives effectively permeated the cell membrane and selectively labeled lysosomes and lipid droplets without causing cytotoxic effects. Furthermore, BODIPY **3** exhibited a significant fluorescence enhancement of 192.5% as the intracellular pH decreased from 7.0 to 4.5, confirming its potential as a bioimaging pH probe. Therefore, these results highlight the potential of these BODIPY derivatives as versatile fluorescence probes for investigating intracellular pH variations and organelle dynamics in living cells through fluorescence imaging.

## Graphical abstract

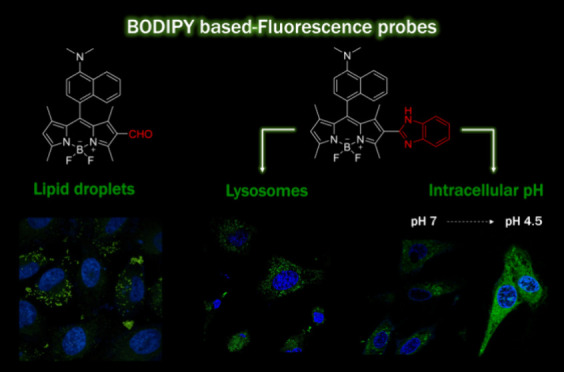

## 1 Introduction

Lipid droplets (LDs) and lysosomes are important organelles in cellular metabolism, playing vital roles in maintaining intracellular homeostasis. LDs not only store and metabolize neutral lipids but also contribute to energy balance, membrane dynamics, and lipid trafficking within cells. Dysfunctional LDs can lead to metabolic disorders and diseases like obesity, fatty liver disease, diabetes, atherosclerosis, and certain types of cancers.^1–3^

Lysosomes are acidic organelles that significantly contribute to the regulation of cellular processes. The optimal functioning of lysosomes is intricately linked to the maintenance of an appropriate intracellular pH. Typically, the intracellular pH hovers within the range of 6.8 to 7.2. However, lysosomes, being the primary acidic compartments in cells, exhibit lower pH values, typically reported between 4 and 7. This acidic environment is crucial for the activation and optimal function of lysosomal enzymes, ensuring the effective degradation of cellular waste. Any dysregulation of lysosomal pH can have profound implications, potentially disrupting cellular processes and contributing to various diseases, including Alzheimer’s and cancer.^4–9^ Additionally, the design of pH sensors to operate in the pH range of 1 to 4 is crucial for studying disorders associated with acidification of the gastrointestinal system, as well as for investigating bacteria such as *Escherichia coli* and *Salmonella* in the highly acidic environment of the stomach.^10^

Fluorescence chemosensors have emerged as indispensable tools for exploring these cellular structures and monitoring intracellular pH fluctuations. Their high sensitivity and ability to provide real-time spatial and temporal information make them invaluable in elucidating the dynamics of organelles within living cells, which significantly contributes to the understanding of complex biological processes.^11–13^ Several organic molecules have been used as fluorophores in this field; however, 3-difluoroborodipyrromethene (BODIPY) derivatives have achieved great notoriety in bioimaging due to their outstanding properties, especially strong absorption, high fluorescence quantum yield, good photochemical stability, low phototoxicity and photobleaching, good cell permeability, and versatile synthesis.^14–19^

Among the strategies to enhance the pH sensitivity of BODIPY fluorophores, the incorporation of piperazine, morpholine, phenol, and aminophenyl groups has been extensively explored, taking advantage of their ability to induce pH-dependent fluorescence through the protonation/deprotonation dynamics.^10,20–23^ Functionalization with a benzimidazole moiety has also been reported.^24–26^ BODIPY derivatives functionalized at the 2-position with benzimidazole heterocycle exhibited enhanced fluorescence under acidic conditions and were successfully applied as lysosomal bioimaging probes.^24,25^ However, these derivatives have not been investigated for tracking intracellular pH variations.

BODIPY derivatives have also been developed for the visualization of lipid droplets.^27–29^ Notably, commercial dyes based on these derivatives include BODIPY 505/515 and BODIPY 493/503. However, their application is limited by issues of specificity, photostability, small Stokes shifts, and background noise. Additionally, careful analysis is required to avoid false-positive fluorescence signals, making the use of appropriate controls essential for accurate data interpretation.^1^ These challenges have driven the development of novel fluorescent probes with improved properties for more reliable and specific LD imaging.

In continuation of the research developed by our group,^25,30–33^ here, we explore the application of two *meso*-dimethylaminonaphthyl-BODIPY-based fluorescent probes, functionalized with formyl group or benzimidazole heterocyclic moiety at position 2, for live cell imaging of intracellular pH, lysosomes, and lipid droplets. Computational and photophysical studies were conducted to evaluate the influence of the chemical environment on the absorption spectra of BODIPY derivatives. *In vitro* studies using confocal microscopy were performed to investigate the internalization of BODIPY derivatives **2** and **3** in HeLa cells, as well as to evaluate the specificity of these compounds for labeling lysosomes and lipid droplets. Additionally, BODIPY **3** was investigated as a fluorescence probe to monitor changes in the intracellular pH within living cells.

## 2 Materials and Methods

### 2.1 General

The synthesis and partial characterization of BODIPY derivatives **1–3** have been reported recently by our research group.^25,30–34^ The complete characterization is presented in the Supporting Information.

### 2.2 Computational Studies

The geometry of BODIPY derivatives **1**, **2**, and **3** (and their respective conjugated acids) was optimized using the density functional theory (DFT) formalism under the ωB97X^35^ approximation with D3BJ long-range corrections^36^ and the Def2-TZVP set of basis functions.^37^ For the initial optimization of the molecular geometries, the solvent effect was incorporated using the C-PCM continuum approach by Barone and co-workers.^38^ The resulting geometries were verified to be true minima of the potential energy surface by the exclusive presence of non-negative vibrational frequencies upon vibrational analysis, which also allowed the estimation of absolute values of the thermochemistry functions (enthalpy and Gibbs energy) at the same level of theory. The absorption spectra of these compounds were initially assessed by using the time-dependent DFT framework (TDDFT) at the same level of theory and solvent configuration used for the geometry optimization routines. All DFT and TDDFT calculations were carried out using the Orca software, version 5.0.3.^39^

Refinement of the absorption spectra was achieved using an adaptation of the protocol established by Cappelli and co-workers.^40^ Each compound was placed at the center of a nanodroplet with a radius of 15.0 Å and containing 90 water molecules or 10 *n*-octanol molecules, using the Packmol software.^41^ Molecular Dynamics (MD) simulation of each nanodroplet was then carried out using the GFN-FF force field by Grimme and Spicher,^42^ a Berendsen thermostat set at 300.0 K for 1.0 ns using an integration step of 1.0 fs, with snapshots of the system’s geometry being collected every 50 steps. During the MD simulation, the nanodroplet was constrained by applying a spherical log-Fermi potential, and long-range solvation effects were taken into account using the analytical linearized Poisson–Boltzmann (ALPB) solvation model, as implemented in the xTB software, version 6.6.1.^43^ Grimme’s sTDA^44^ approach was then used to calculate the absorption of each nanodroplet in 200 frames of the MD trajectory, sampled randomly using an in-house developed Python script. In order to obtain the final sTDA for each compound and medium, the absorption peaks of each of the 200 sampled frames were broadened using a Gaussian spread of 10 nm (full width at half height) and weighted by their relative energy, using Boltzmann’s distribution:^45^

$$\omega_{i}=\frac{e^{-\Delta E_{i}/kT}}{\sum_{j}e^{-\Delta E_{j}/kT}}$$ 1

where *w*_*i*_ is the weight attributed to frame *i*, Δ*E*_*i*_ is the energy of frame *i* (relative to the lowest energy frame in the set), *T* is the temperature (set at 300.0 K), and *k* is Boltzmann’s constant.

### 2.3 pH-Dependent Absorption and Emission of BODIPY Derivatives **2** and **3**

Absorption spectra were recorded on a Shimadzu UV-2600i spectrophotometer. Fluorescence emission spectra were recorded by using a Horiba–Jobin–Yvon Fluorolog-322 fluorimeter and a FluoroMax-4 (HORIBA) spectrofluorometer. The measurements were conducted with 3 nm slits and an integration time of 0.5 s. The excitation wavelength was systematically varied in 5 nm increments, and the emission spectra were collected over a wavelength range of 476 to 800 nm. The study was carried out in a buffer pH = 4 (20 °C), citric acid/sodium hydroxide/sodium chloride solution (Honeywell, Fluka), and a Phosphate-buffered saline (PBS) buffer pH = 7.4 solution.

### 2.4 Absorption and Fluorescence Spectral Changes of BODIPY 3: pH Response and p*K*_a_ Determination

The evaluation of BODIPY **3** as a pH probe was performed in water at a pH range from 2.2 to 8.7. The solutions with different pH values were prepared in Milli-Q water by adding HCl (1 M) or NaOH (1 M), and the pH was determined with a pH meter. A stock solution of the BODIPY derivative was prepared in DMSO (10 mM) and then diluted in the pH solutions to a final concentration of 10 μM. Absorption and fluorescence spectra were collected using a Biotek Synergy H1 plate reader (λ_ex_ = 480 nm). The data were fitted to a sigmoidal dose–response curve using OriginPro 8.5 software. The p*K*_a_ was determined from the data by finding the midpoint (l­(x_0_)) of the sigmoidal curve eq 2 (Table 1):^46^

**Table 1:** pKa Determination of BODIPY Derivative **3** Based on the Sigmoidal Curve

**model**	DoseResp
equation	$=A_{1}+\frac{A_{1}-A_{2}}{1+10^{\left(\log\left(x_{0}\right)-x\right)*p}}$
*A*_1_	0.0052 ± 0.0004
*A*_2_	1.08597 ± 0.00664
log(*x*_0_)	2.86045 ± 0.0072
*p*	–1.61012 ± 0.003258
reduced chi-sqr	8.23305 × 10^–7^
Adj. *R*-square	0.99999

### 2.5 Cell Culture

Human cervical cancer (HeLa) cells were grown in Dulbecco’s modified Eagle’s medium (DMEM) (Gibco, Thermo Fisher Scientific, Waltham, MA, USA) supplemented with 10% fetal bovine serum (FBS) and 1% of penicillin-streptomycin, at 37 °C in a humidified incubator with a 5% CO_2_ atmosphere.

### 2.6 Cell Viability

HeLa cell viability was determined by the resazurin assay.^47,48^ Furthermore, 10^4^ cells per well were seeded in 96-well plates and incubated overnight at 37 °C in a 5% CO_2_ atmosphere. Then, cells were incubated with BODIPY derivatives **2** and **3** at concentrations ranging from 3 to 100 μM for 24 h. Afterward, cells were treated with a resazurin reagent (1:100) and incubated for 4 h in the dark. The fluorescence intensity was measured in the Biotek Synergy H1 microplate reader, and the cell viability values were calculated considering the control (nontreated cells) as 100% viability, using eq 3. The blank represents the fluorescence mean value of the cell culture medium. Data were represented as mean ± SEM­(standard error of the mean) from triplicate samples of two independent experiments.

$$\mathrm{cell}\;\mathrm{viability}\left(\%\right)=\frac{\mathrm{Fluo}\left(\mathrm{treated}\;\mathrm{cells}\right)-\mathrm{Fluo}\left(\mathrm{blank}\right)}{\mathrm{Fluo}\left(\mathrm{control}\right)-\mathrm{Fluo}\left(\mathrm{blank}\right)}\times 100$$ 3

### 2.7 Subcellular Localization

#### 2.7.1 Lipid Droplet Colocalization Assay

The protocol described by the manufacturer of the commercial LipidSpot Lipid Droplet Stains probe (Biotium, San Francisco Bay Area, CA, USA) was followed to perform the lipid droplet colocalization assay. First, to induce lipid droplet formation in cells, oleic acid complexed with BSA was used. HeLa cells were seeded at a density of 2 × 10^4^ cells per well in DMEM in glass bottom dishes and incubated overnight at 37 °C in a 5% CO_2_ atmosphere. Oleic acid and defatted BSA solutions were prepared for further complex formation. The oleic acid was diluted to 150 mM in 50% ethanol by mixing 47 μL of oleic acid with 953 μL of 50% ethanol. The mixture was vortexed and stored at 4 °C. A 100 mg/mL BSA solution was prepared by dissolving the defatted BSA in dH_2_O and stored at −20 °C. Then, the oleic acid solution was resuspended by heating to 37 °C, and equal volumes (20 μL) of 150 mM oleic acid and 100 mg/mL defatted BSA in dH_2_O were combined. The mixture was incubated at 37 °C for 1 h. This oleic acid/BSA complex was diluted 1:150 in growth medium for a final concentration of 500 μM oleic acid, and the cells were incubated with this solution overnight at 37 °C. Cells were incubated at 37 °C with a 1:1000 solution of LipidSpot for 30 min to label lipid droplets in the cell cytoplasm, with BODIPY **2** at a concentration of 40 μM (a stock solution of 10 mM in DMSO diluted with DMEM) for 20 min and, finally, with a 1:1000 solution of Hoechst 33342 (Abcam, Cambridge, UK) for 10 min to stain the cell nucleus. The cells were then imaged by confocal microscopy (LSM 780 Zeiss, Oberkochen, Germany) with a 63× oil immersion objective lens, using a 405 nm laser for Hoechst 33342, a 488 nm laser for the BODIPY **2**, and a 633 nm laser for LipidSpot.

#### 2.7.2 Lysosome Colocalization Assay

HeLa cells were seeded at a density of 2 × 10^4^ cells per well in DMEM in glass bottom dishes and incubated overnight at 37 °C in a 5% CO_2_ atmosphere. Then, cells were washed two times with PBS and treated with BODIPY **3** at a concentration of 40 μM (a stock solution of 10 mM in DMSO diluted with DMEM) and with an 85 nM solution of LysoTracker Deep Red (Invitrogen, Waltham, MA, USA) for 1 h to stain lysosomes in the cell cytoplasm. Afterward, the cell nucleus was stained with a 1:1000 solution of Hoechst 33342 (Abcam, Cambridge, UK) for 10 min. The fluorescence cell images were collected with confocal microscopy (LSM 780 Zeiss, Oberkochen, Germany) under a 63x oil-immersion objective lens, using the 405 nm laser for Hoechst 33342, a 488 nm laser for the BODIPY **3**, and a 633 nm laser for LysoTracker Deep Red.

### 2.8 Quantitative Analysis of Colocalization Assay

Signal thresholds were determined using samples incubated with BODIPY derivative **2** or Lipid droplet and BODIPY derivative **3**. Colocalization analysis was carried out using ImageJ with plugins JACoP^49,50^ and R statistical Language V4.2.2 (R Core Team, Vienna, Austria).^51^ The measurements were repeated in at least three independent images for three different cells. Costes’ automatic threshold was used on the images of each sample to minimize the contribution of sample noise.^52^ Pearson’s correlation coefficient (*r*), where *R*_*i*_ and *G*_*i*_ represent red and green channel intensity in each pixel, and *R̅* and *G̅* are red and green channel average intensity, was calculated to demonstrate positive spatial correlation (eq 4). The average and standard deviation of the results were calculated for each condition and results.

$$r=\frac{\sum_{i}\left(R_{i}-\mathrm{R̅}\right)\times \left(G_{i}-\mathrm{G̅}\right)}{\sqrt{\sum_{i}{\left(R_{i}-\mathrm{R̅}\right)}^{2}\times \sum_{i}{\left(G_{i}-\mathrm{G̅}\right)}^{2}}}$$ 4

### 2.9 Intracellular pH Assay

The buffer solutions were prepared in Milli-Q water with 20 mM sodium chloride, 135 mM potassium chloride, 20 mM glucose, 1 mM magnesium sulfate, 20 mM HEPES, 1 mM calcium chloride, and 10 μM nigericin sodium salt (Sigma-Aldrich, St. Louis, Missouri, EUA). The pH of each solution was adjusted with KOH (1 M) or HCl (1 M) to obtain pH values of 4.5 and 7. Nigericin is an H^+^/K^+^ ionophore used to equilibrate the intracellular pH with the extracellular pH.^53^

For the intracellular pH bioimaging, HeLa cells were seeded at a density of 2 × 10^4^ cells/well in growth medium in glass bottom dishes and incubated overnight at 37 °C in a 5% CO_2_ atmosphere. Then, cells were incubated with nigericin buffer solutions at different pH values for 20 min. After, cells were washed two times with PBS and treated with 20 μM of BODIPY derivative **3** for 30 min. Then, cells were stained with a commercial probe for the nucleus (Hoechst 33342, Thermo Scientific) at a dilution factor of 1000× for 10 min. The cells were then imaged by confocal microscopy (LSM 780 Zeiss, Oberkochen, Germany) with a 63× oil immersion objective with a 405 nm laser for Hoechst 33342 (λ_em_ = 447 nm, blue channel), a 488 nm laser (λ_em_ = 530 nm, green channel), and a 561 nm laser (λ_em_ = 610 nm, red channel), both for BODIPY derivative **3**. The average fluorescence intensity of each channel was analyzed by R statistical Language V4.2.2 (R Core Team, Vienna, Austria), with which we calculated the average absolute fluorescence of the images per cell and color channel at each pH studied. The comparison was performed by keeping the laser gain constant between images. The total increase (gain) in fluorescence between the pH conditions was calculated through eq 5:

$$\mathrm{Fluorescence}\;\mathrm{gain}\left(\%\right)=\frac{\mathrm{Fluorescence}\left(\mathrm{pH}\;4.5\right)-\mathrm{Fluorescence}\left(\mathrm{pH}\;7\right)}{\mathrm{Fluorescence}\left(\mathrm{pH}\;7\right)}\times 100$$ 5

## 3 Results and Discussion

### 3.1 Synthesis and Structural Characterization of BODIPY Derivatives **1**–**3**

BODIPYs **2** and **3** are based on the tetramethyl-BODIPY core functionalized with an electron-donating *N*,*N*-dimethylaminonaphthyl group at the 8-position (*meso* position), and formyl and benzimidazole electron-withdrawing groups at the 2-position, respectively. The synthesis and partial characterization of BODIPY derivatives **1**–**3** have been reported previously by our research group.^30,34^

Briefly, precursor **1** was synthesized through condensation, oxidation with DDQ, and boron chelation, yielding a symmetrical *meso*-substituted *N,N*-dimethylaminonaphthyl-BODIPY. Subsequently, via the Vilsmeier–Haack reaction formylation of precursor **1** with DMF/POCl_3_, we obtained the formylated BODIPY derivative **2**. Finally, derivative **3**, functionalized with a benzimidazole moiety, was synthesized through the condensation of compound **2** with *o*-phenylenediamine (Scheme 1).

**Scheme 1:**
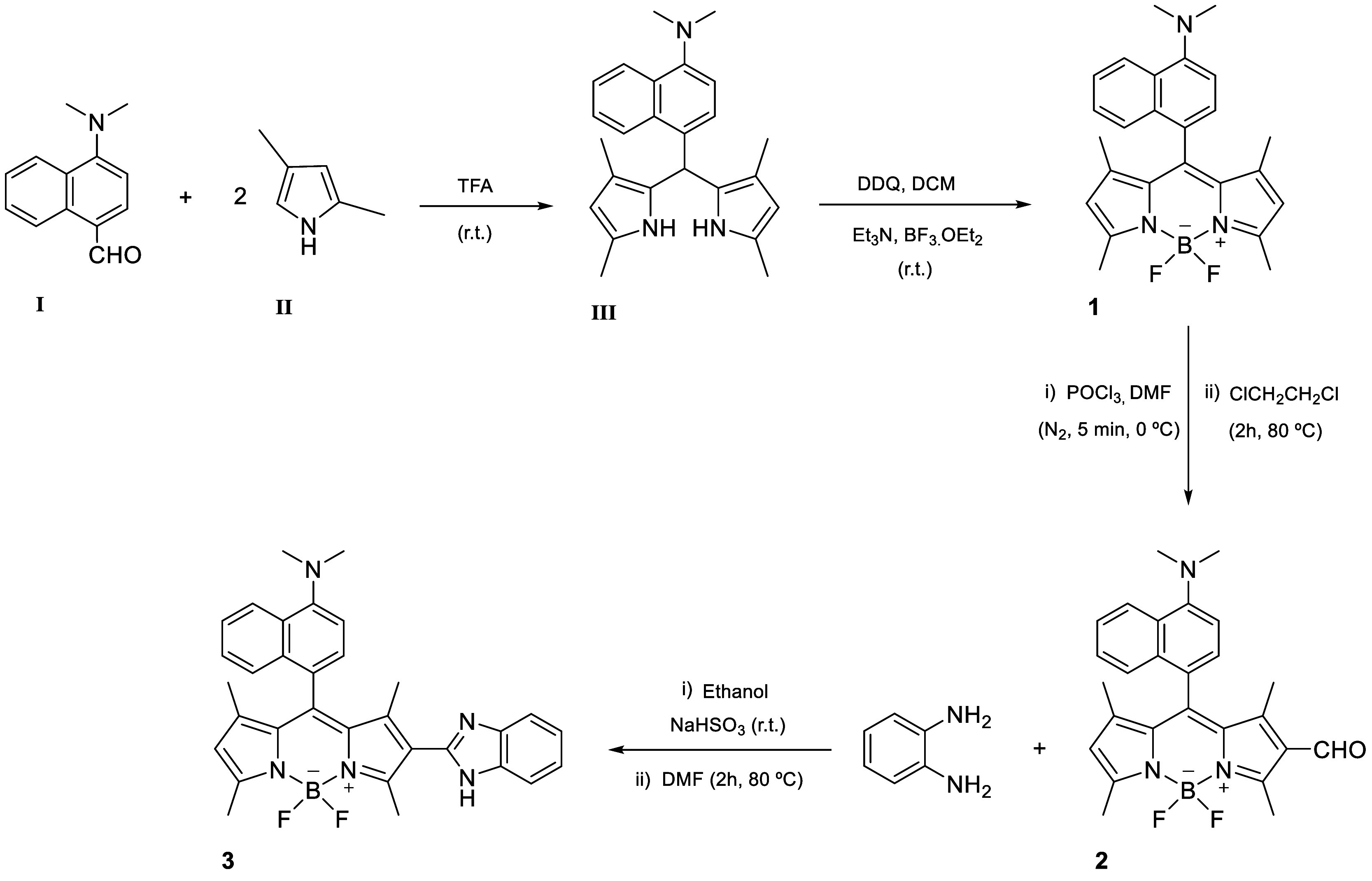
Synthetic Approach of BODIPY Derivatives **1**–**3** ^30,34^

Identification of the three BODIPY derivatives **1**–**3** was performed through ^1^H and ^13^C nuclear magnetic resonance (NMR) spectroscopy, as well as high-resolution mass spectrometry (HRMS).^30,34^ Furthermore, the compounds were analyzed by Fourier transform infrared spectroscopy with attenuated total reflectance (FTIR-ATR) (see SI), revealing distinctive spectral features such as the C=O band at approximately 1662 cm^–1^ in the spectrum of the BODIPY derivative **2**. These techniques collectively confirmed the proposed molecular structures.

All compounds displayed the typical absorption and emission features of the BODIPY chromophore with absorption maxima in the range 494–512 nm and fluorescence emission maxima between 509 and 514 nm. The functionalization of the BODIPY core with a benzimidazole moiety induced a slight shift of the absorption and emission maxima to longer wavelengths compared to its precursors, BODIPY **1** and **2**.^34^ Moreover, the relative fluorescence quantum yield of BODIPY **3** was found to be lower relative to compounds **1** and **2**, yet this feature can be particularly advantageous considering activatable fluorescent imaging probes, in which its fluorescence emission is only triggered under specific conditions, for instance in response to biomolecules, microenvironment, or reactive species.^54^

### 3.2 Photophysical Characterization of BODIPY Derivatives **2** and **3**

Further insights on the acid–base behavior, solvation, and photophysical characteristics of compounds **2** and **3** were gathered from a combination of computational and experimental assays. The p*K*_a_ of these two compounds was estimated using a simplified variation of the thermochemistry-based techniques championed by Olsen et al.^55^ and Frau et al.^56^ As detailed in the Supporting Information, the geometries of the neutral and acidic forms of 14 organic compounds with either the benzimidazole moiety or with one amine group attached to an aromatic system were calculated at the same level of theory of the BODIPY derivatives under study, and a linear relationship was established between their p*K*_a_ and the variation of enthalpy at 298 K upon protonation. This linear relationship was then used to estimate the p*K*_a_ of the three BODIPY derivatives, presented in Table 2. These results show that the p*K*_a_ values of compounds **1** and **2** are close to those of other aromatic amines, such as 1-naphtylamine (p*K*_a_ = 3.92)^57^ and *N*-methylnaphthalen-1-amine (p*K*_a_ = 3.67).^58^ As expected, compound **3** has a more involved acid–base behavior due to the availability of two basic nitrogen atoms: one at the benzimidazole moiety (hereafter referred to as position B) and the other at the amino group of the *N,N*-dimethylaminonaphthyl moiety (position A). The thermochemistry data summarized in Table 2 reveals that protonation at the benzimidazole moiety is more favorable, leading to an estimated p*K*_a__2_ of 5.91, which is in line with other known benzimidazoles, such as benzimidazole (p*K*_a_ = 5.48)^59^ and 2-methyl-benzimidazole (p*K*_a_ = 6.19). Protonation at the amino-naphthyl moiety of compound **3** is energetically unfavorable, but a minute population of this form may be present at about pH = 4. The diprotonated form of compound **3** has an estimated p*K*_a_ of 2.44.

**Table 2:** Absolute Energies, Calculated at the ωB97X-D3BJ/Def2-TZVP Level of Theory and Including Zero-Point Energy Corrections, Variation of Enthalpy for the Deprotonation of Each Acidic Form of BODIPY Derivatives **1** to **3** to Their Neutral Form, and Estimated pKa Values for Each Form

compound	species	*E*/*E*_h_	Δ*H*/kJ mol^–1^	estimated p*K*_a_
1	neutral	–1279.73642		
1	acid (A)	–1280.18255	+97.22	3.51
2	neutral	–1393.13915		
2	acid (A)	–1393.58494	+96.48	3.47
3	neutral	–1393.13915		
3	acid (A)	–1659.16839	+139.75	3.42
3	acid (B)	–1659.18364	+96.03	5.91
3	diprotonated	–1659.62806	+235.44	2.44

The absorption spectra of BODIPY derivatives **2** and **3**, as well as that of their respective conjugated acids, were predicted using Time-Dependent Density Functional Theory (TDDFT) calculations in which the effect of the solvent was addressed using an implicit solvent (continuum method). The results shown in Figure 1 demonstrate that TDDFT systematically failed to predict the characteristic BODIPY absorption band at about 500 nm.^60^ While the absorption bands of compounds **2** and **3** are centered in the region between 500 and 525 nm, TDDFT calculations offset the location of the lowest energy absorption peaks by about 100 nm. In order to attain better predictions regarding this band, Molecular Dynamics (MD) simulations of BODIPY derivatives **2** and **3** in water were carried out, and the sTDA-predicted absorption spectra of 200 randomly selected frames of the MD trajectory were weighted using a Boltzmann distribution. The results depicted in Figure 1 suggest this approach outperformed TDDFT, although with varying accuracy depending on the specific molecular system. Indeed, the absorption spectra of compound **2** in its neutral form shows a distinct absorption band at 505 nm (Figure 1a), TDDFT wrongly predicts this transition to take place at 395 nm, while MD/STDA predicts this band to be centered at 488 nm, which is in very good agreement with the experimental observation. A similar scenario is also observed for the neutral form of compound **3** (Figure 1c), for which TDDFT predicts the lowest energy absorption band to be centered at 411 nm. On the other hand, MD/sTDA predicts an intense absorption band centered at 515 nm, excellent agreement with the experimentally determined value of 521 nm, despite the presence of a minor artifact at about 600 nm and an over estimation of the intensity of the band at about 400 nm, which appears as a shoulder in the experimental spectrum (Figure 1c).

**Figure 1:**
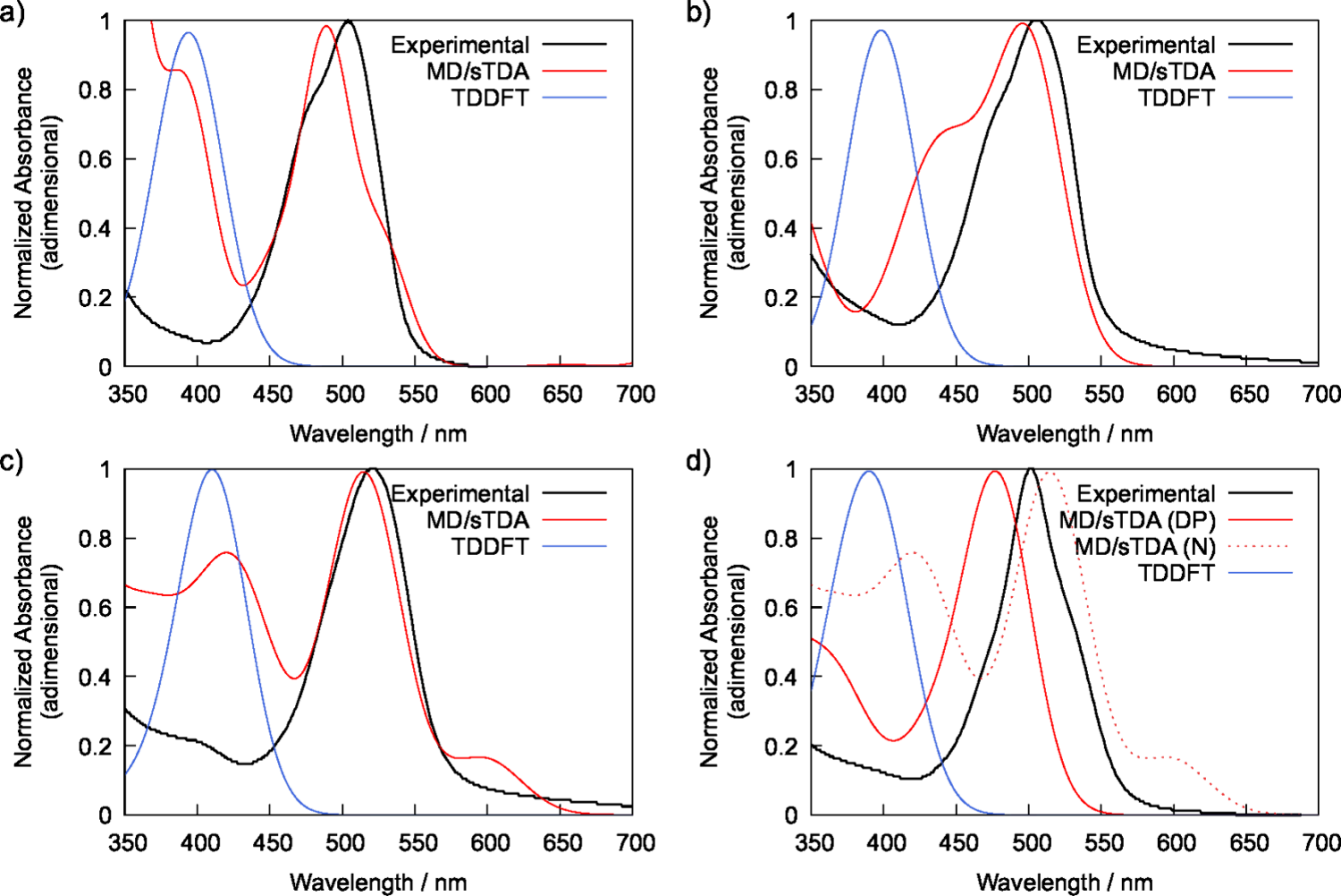
Experimental absorption spectra of BODIPY derivatives and their respective theoretical predictions using standard TDDFT calculations as well as ensemble-weighted MD/sTDA spectra in water: (a) compound **2** in buffer at pH = 7.4; (b) compound **2** in buffer at pH = 4 and the theoretical predictions for its conjugated acid; (c) compound **3** at pH = 7.4, and; (d) compound **3** at pH = 4 and the theoretical predictions for its diprotonated (DP) acidic form, with the predicted spectrum of its neutral (N) form depicted in dashed lines. In all theoretical predictions a Gaussian spread of 55 nm (fwhm) was applied to each predicted excitation energy in order to match the width of the experimental spectra.

The predicted values of p*K*_a_ for compounds **2** and **3** (Table 2) suggest that at pH = 4, each of these compounds is spread over multiple species. Indeed, some crude equilibrium calculations (detailed in the Supporting Information) suggest that at pH = 4, about 60% of compound **2** remains in its neutral form, while for compound **3** about 64% is present in its neutral form and about 35% is present in its diprotonated form, with the two intermediate acid species making up for the remaining 1% of the population. This hinders the comparison between the experimental spectra and the corresponding predictions. Despite this, MD/sTDA yielded considerable accuracy in predicting the lowest energy absorption band of this species in acidic media. As shown in Figure 1b, MD/sTDA predicts the location of the maximum absorption band of the conjugated acid of compound **2** at 496 nm (experimentally determined at 505 nm), while TDDFT predicted this band to be centered at 398 nm. A similar behavior is also observed for the conjugated acid of compound **3**, for which the experimental peak is registered at 501 nm (Figure 1d), with MD/sTDA predicting the maximum absorption in the visible range to take place at 477 nm for the diprotonated acid (DP), and at 515 nm for its neutral (N) form. Although not accurate enough to enable a semiquantitative evaluation of the speciation of compound **3**, the MD/sTDA results remain in very good agreement with the observed reality.

Apart from outperforming TDDFT at predicting the absorption spectra of compounds **2** and **3**, the MD/sTDA approach also provided, as a side product, some insights into the solvation of these compounds by analyzing the trajectory of the MD simulations. Figure 2 shows the spatial distribution function (SDF) of water’s oxygen and hydrogen atoms around compounds **2** and **3**, as well as the protonated form of compound **2** and the DP form of compound **3**. The results from the MD simulations highlight the strong affinity between water and the BODIPY moiety. This is particularly evident in the neutral form of compound **2** (Figure 2a), where it is possible to infer the higher likelihood of finding the water molecules hovering above and below the BODIPY plane, with one hydrogen atom preferentially pointing toward the fluorine atoms. Upon protonation, a secondary water shell forms around the ammonium group (Figure 2b), stabilized by the Coulombic attraction and formation of hydrogen bonds between the ammonium group and the lone pairs of water’s oxygen atom. The presence of the benzimidazole moiety in the BODIPY derivative **3** poses a secondary location of preferential solvation, with water molecules being attracted to the vicinity of these group’s nitrogen atoms, although with no discernible preferential orientation, as suggested by the absence of localized concentration of hydrogen atoms in Figure 2c. As expected, this region around the benzimidazole moiety becomes more defined in the DP form of compound **3**, which shows three regions were water molecules seem con concentrate: the BODIPY core and the ammonium group, as well as the protonated benzimidazole region, were the preferential orientation of the water molecules, with their hydrogen atoms pointing away from the imidazole subregion of the benzimidazole moiety is very well-defined in Figure 2d.

**Figure 2:**
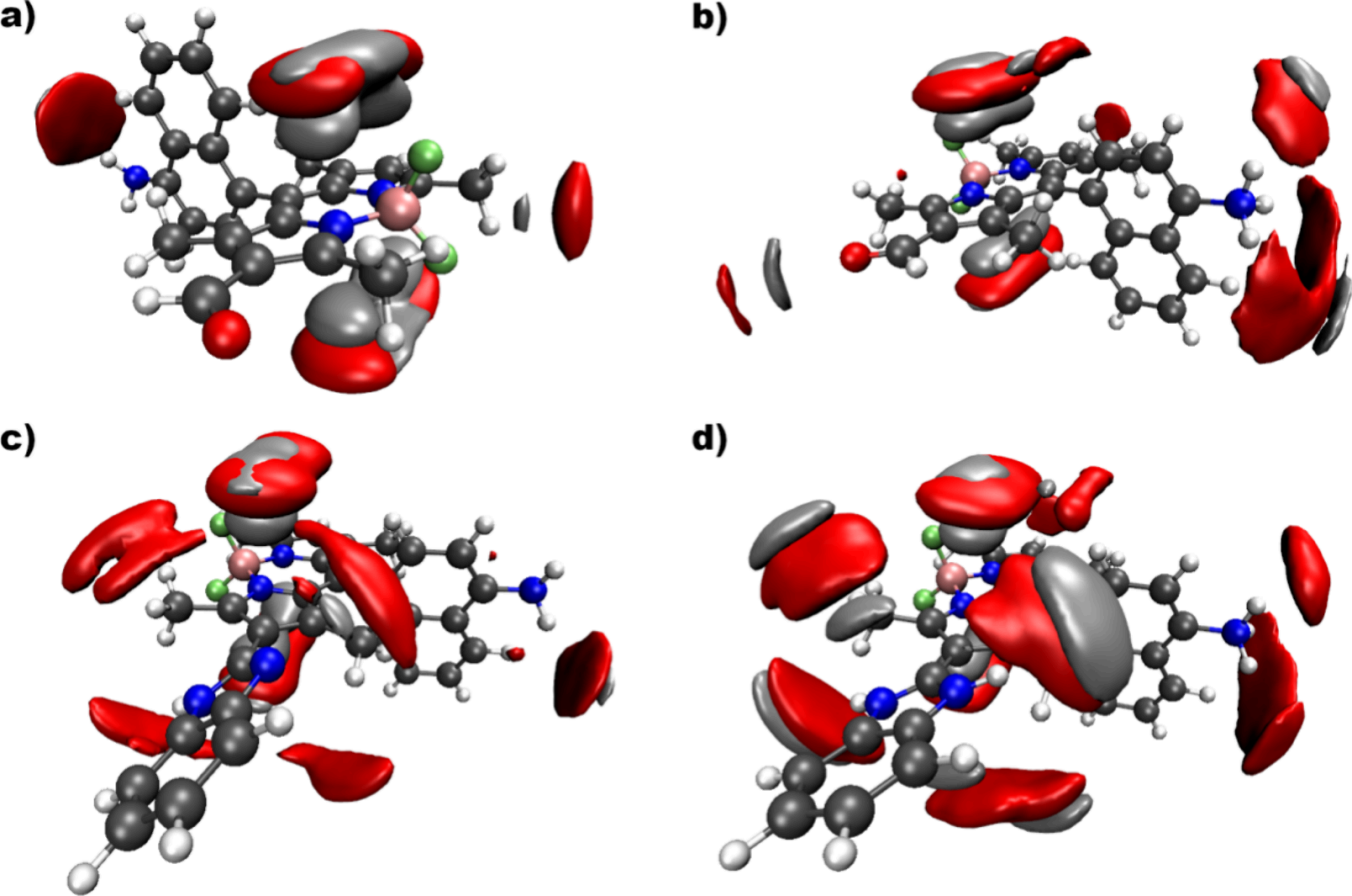
Representation of the spatial distribution function (SDF, isosurface at 40 nm^–3^) of the oxygen (red) and hydrogen (gray) atoms of the water molecules solvating BODIPY derivatives **2** (a) and its conjugated acid (b), as well as **3** (c) and its diprotonated acid form (d).

In order to better understand the behavior of these compounds in a more hydrophobic medium, the same MD/sTDA protocol was carried out in the presence of *n-*octanol. Before discussing the effect of the solvent on the absorbance spectra, we first analyzed the SDF profile of the octanol-solvated BODIPY derivatives. The most prominent feature of these profiles is the consistent concentration of butanol’s hydroxyl group(s) in the vicinity of the BODIPY moiety, as exemplified in Figure 3a for compound **3**, which is also representative of the SDF observations for compound **2** in its neutral and protonated forms. On the other hand, the SDF profile of the octanol-solvated compound **3** in its diprotonated form reveals some strong attraction of the solvent’s hydroxyl group toward the protonated benzimidazole moiety, as shown in Figure 3b. It is noteworthy that the only significant concentration of octanol’s carbon atoms takes place in the outer regions of the “hydroxyl-BODIPY-hydroxyl” sandwich. The relatively small footprint of this SDF (cf. Figure 3) suggests that this is the preferred orientation of octanol’s C1, and that the rest of the solvent molecule moves in a relatively free manner during the MD simulation. Hence, the results from the MD simulations point toward a more labile interaction between these BODIPY derivatives and a more hydrophobic solvent, compared to what is observed in aqueous medium.

**Figure 3:**
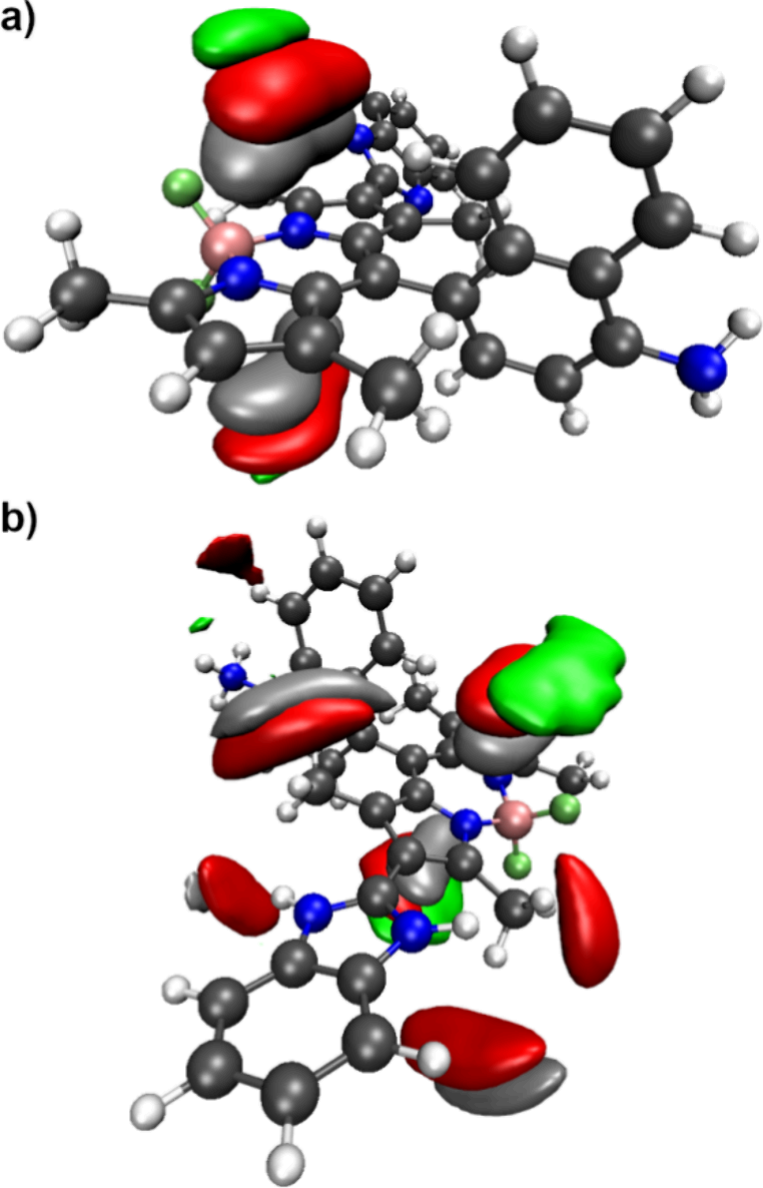
Representation of the SDF of oxygen (red), hydrogen (gray), and carbon (green) atoms of the octanol molecules solvating BODIPY derivative **3** (a) and its diprotonated acid form (b). The isosurface marks the 40 nm^–3^ threshold for oxygen and hydrogen and the 15 nm^–3^ threshold for the carbon atoms.

The insights acquired from observing the solvation of the BODIPY derivatives in octanol help to shed some light on the MD/sTDA predicted absorption spectra of these compounds in its neutral and acid forms. The results shown in Figure 4 suggest that the position of the maximum absorption band of compound **2** does not shift significantly when transitioning to a more lipophilic medium (Figure 4a). It is worth noting that in aqueous medium, the MD/sTDA predictions overstated the intensity of this compound’s absorption in the 300 to 400 nm region. Although this problem seems to be ameliorated in *n-*octanol, a new artifact appears in the 550 to 600 nm region. This artifact, however, takes the form of a relatively low intensity absorption band, suggesting that the absorption spectra in both media are qualitatively the same. As for the conjugated acid of compound **2**, the results shown in Figure 4b show a broadening of the absorption band at about 500 nm, but the overall result does not support the hypothesis of a significant solvatochromic effect for this species.

**Figure 4:**
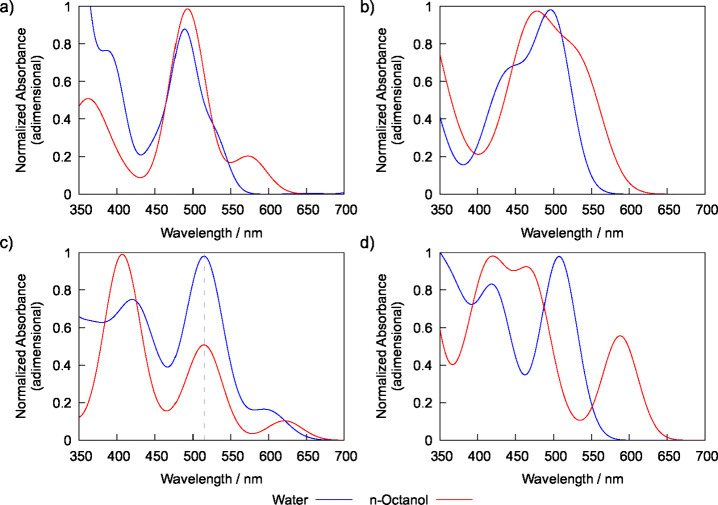
Predicted absorption spectra, in aqueous medium and in *n*-octanol, of BODIPY derivative **2** (a), and its conjugated acid (b), as well as of derivative **3** (c) and its di-protonated corresponding conjugated acids (b and d, respectively) using the ensemble-weighted MD/sTDA protocol. A Gaussian spread of 55 nm (fwhh) was applied to each predicted excitation energy.

On its turn, the results from MD/sTDA calculations show considerable differences for compound **3** with respect to the changing solvent. In this regard, it is important to diagnose potential artifacts that may arise from the calculation. Considering predicted absorption spectra depicted in Figure 4c with the experimental one displayed in Figure 1c, one notices that the most intense band of the predicted spectrum in *n-*octanol corresponds lies in the same region has the experimentally observed low-intensity shoulder at 400 nm, suggesting this band may represent an even greater overestimation of the signal’s intensity in *n-*octanol, when compared with the predictions for the aqueous solution. Furthermore, the weak absorption band observed at 625 nm is most likely related to the spurious band predicted in aqueous medium at about 600 nm. Thus, the only remaining signal in the predicted spectra displayed in Figure 4c relates to the experimentally observed peak at 521 nm. In both solvents, the MD/sTDA prediction is located at 515 nm (dashed gray line in Figure 4c), thus excluding the possibility of a solvatochromic effect.

The MD/sTDA predictions for the DP form of compound **3** in *n-*octanol show a substantial deviation from those carried out in water, as shown in Figure 4d. The former consists mainly of three absorption bands located at approximately 410, 460, and 590 nm. By analogy to the MD/sTDA predictions in aqueous solution, the band located at 410 nm is present in the predictions for both solvents. As discussed above, this is likely an overestimation of the intensity of a low-intensity transition that takes place in these BODIPY derivatives, yielding a low-intensity band or shoulder in the 400 nm region. Indeed, such signal is absent from the experimental spectra recorded for compound **3** and pH = 4 (Cf. Figure 1d), which may be due either to the speciation of compound **3** in the experimental conditions, or to a possible reduction of this transition’s signal in the highly charged DP form of compound **3**. The band at 590 nm may correspond to the same artifact that is observed for the MD/sTDA predictions for these compounds (discussed above), although its location at lower wavelength values and medium intensity are distinctive features when compared, for example, with the artifacts observed in the 600 to 650 nm region in Figure 4c. On its turn, the central band of the DP form of compound **3** in *n*-octanol is located at about 460 nm and corresponds to a blueshift of the 477 nm band observed in water. This brings forth the hypothesis that compound **3** may have different fluorescence signatures depending on the lipophilicity of the medium.

Additionally, the absorption and fluorescence properties of BODIPY **3** were examined across a pH range of 2.2 to 8.7 (Figure 5 and Figure S3). As shown in Figure 5a, decreasing the pH led to a gradual decrease in the absorption band centered at 520 nm, alongside the emergence of a new, blue-shifted band at 500 nm, in line with the MD/sTDA predictions depicted in Figure 1c,d. Fluorescence measurements (Figure 5b) revealed a remarkable enhancement in emission intensity as the pH decreased from 8.7 to 2.2, with a 426-fold increase at the emission maximum. Notably, a 55-fold enhancement was observed when the pH was reduced from 4 to 2.2, whereas a more subtle 8-fold increase occurred between pH 8.7 and 4 (Figure S3). The pH-dependent fluorescence response of BODIPY **3** was further analyzed by plotting the fluorescence intensity at 520 nm against pH. The resulting curve was fitted to a sigmoidal model, yielding a p*K*_a_ value of 2.86 (inset Figure 5b), consistent with the behavior of a compound undergoing diprotonation in acidic conditions. These experimental findings align well with previous computational predictions. The experimentally determined p*K*_a_ value of 2.86 for BODIPY **3** is in good agreement with the *in silico* prediction of 2.44 for the diprotonated form, as estimated using a thermochemistry-based approach. This correlation supports the assignment of the dominant protonation event to the benzimidazole moiety, which was predicted to have a higher p*K*_a_ (5.91), while the lower experimental p*K*_a_ likely reflects the subsequent protonation at the amino-naphthyl group.

**Figure 5:**
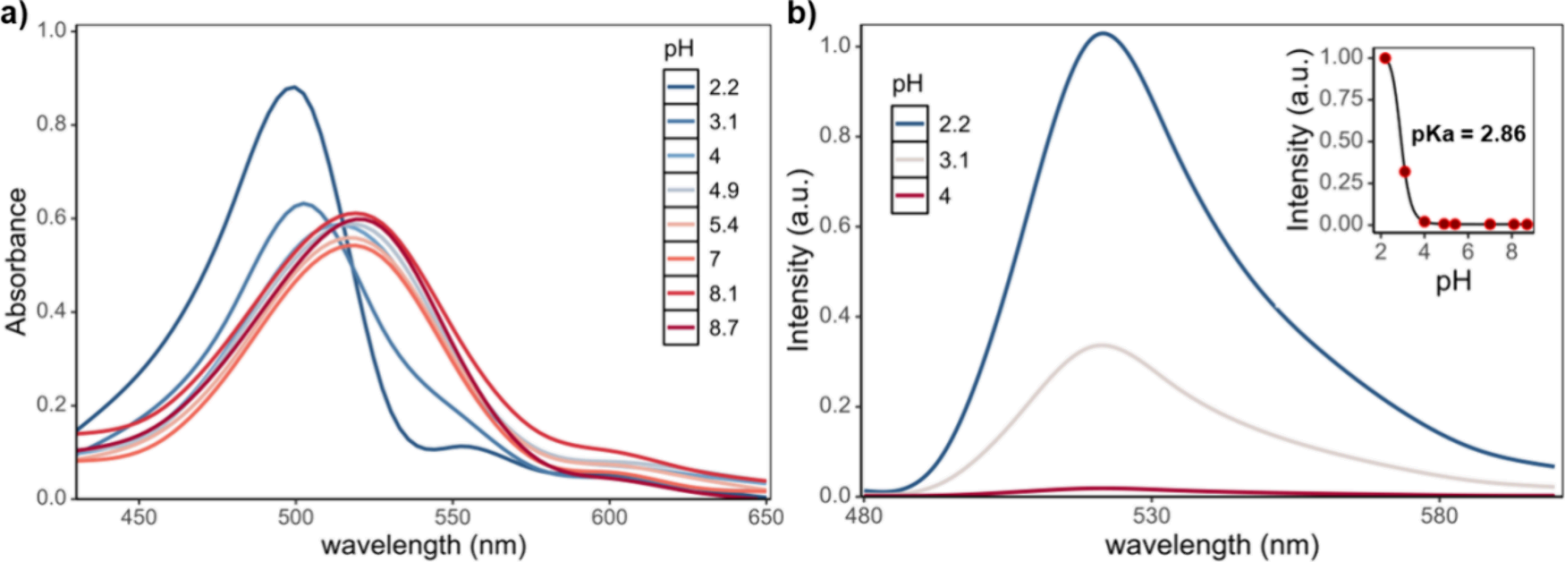
Absorption (a) and fluorescence (b) spectra of BODIPY **3** at different pH values. Inset: sigmoidal curve fitting of the fluorescence intensity at 520 nm as a function of pH.

Finally, the two-dimensional (2D) fluorescence spectra of compounds **2** and **3** in buffer pH = 7.4 and buffer pH = 4 are depicted in Figure **6**. At pH = 7.4, compound **2** exhibits some weak fluorescence in the 700 to 750 nm region (Figure 6a). These signals are absent from the 2D fluorescence spectrum of compound **2** in a more acidic medium (Figure 6b), possibly due to the decreased likelihood of this transition to take place in the charged molecule, leaving only the signal corresponding to local excitation (LE). A similar picture emerges from the 2D fluorescence spectrum of compound **3**. At pH = 7.4 this compound is in its neutral form and CT transitions take place, leading to emission in the 650 to 750 nm region (Figure 6c). On the other hand, at pH = 4, the overall positive charge of the system prevents CT to take place, leading to a fluorescence spectrum highlighting the LE signal (Figure 6d).

**Figure 6:**
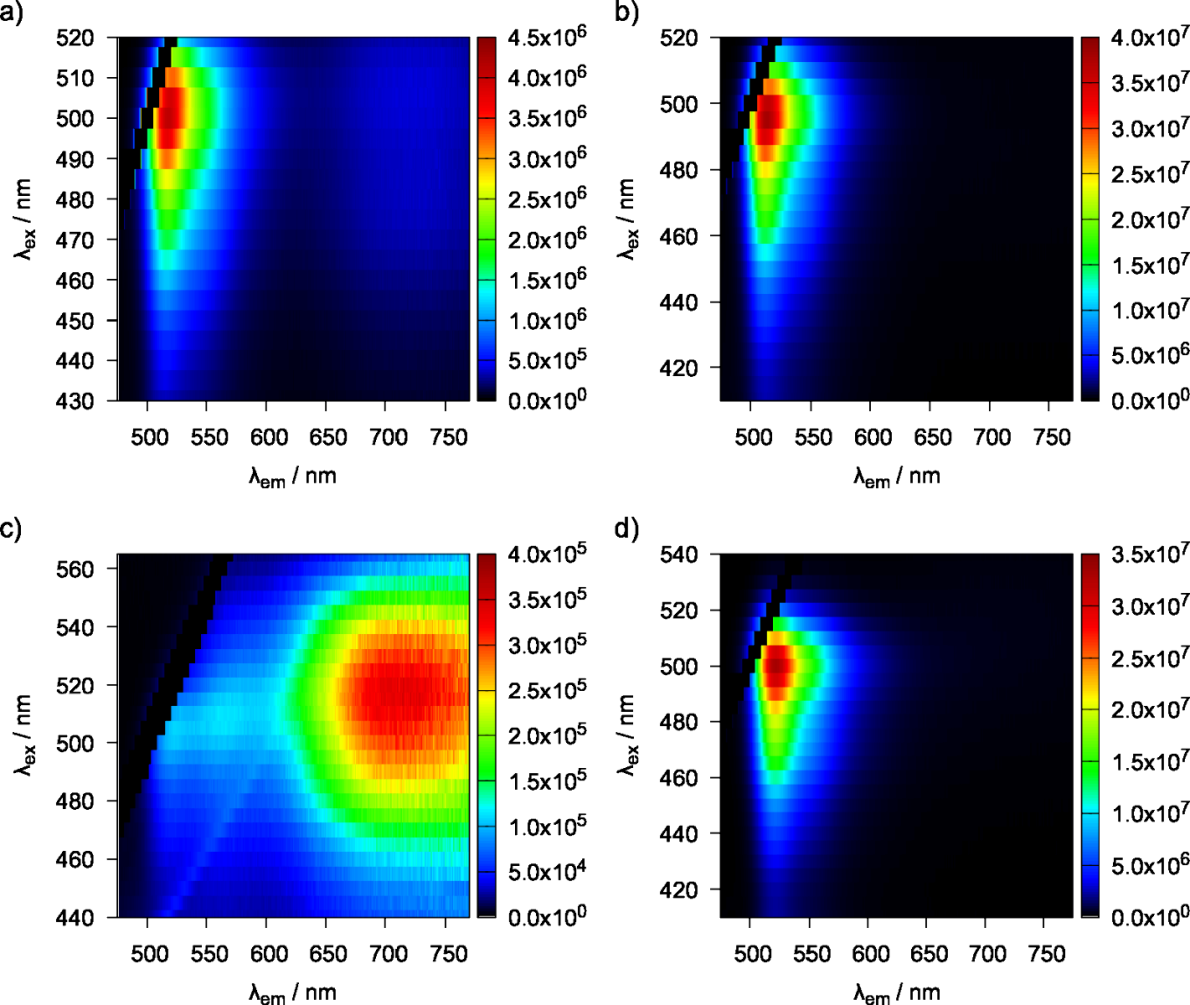
Two-dimensional fluorescence spectra of BODIPY derivative **2** at pH = 7.4 (a); and at pH = 4 (b); and of BODIPY derivative **3** at pH = 7.4 (c); and in buffer at pH = 4 (d). The black line near the top left corner is due to removal of the excitation peak values.

### 3.3 Cell Viability

Prior to cell imaging, BODIPYs **2** and **3** cytotoxicity was evaluated in HeLa cells using a resazurin assay. HeLa cells were treated with different concentrations of the compounds (3–100 μM), and, after 24 h of incubation, the cell viability was determined. As shown in Figure 7, cell viability remained greater than 95% with concentrations of 50 μM after 24 h. Thus, BODIPYs **2** and **3** proved to be biocompatible and appropriate for further bioimaging experiments.

**Figure 7:**
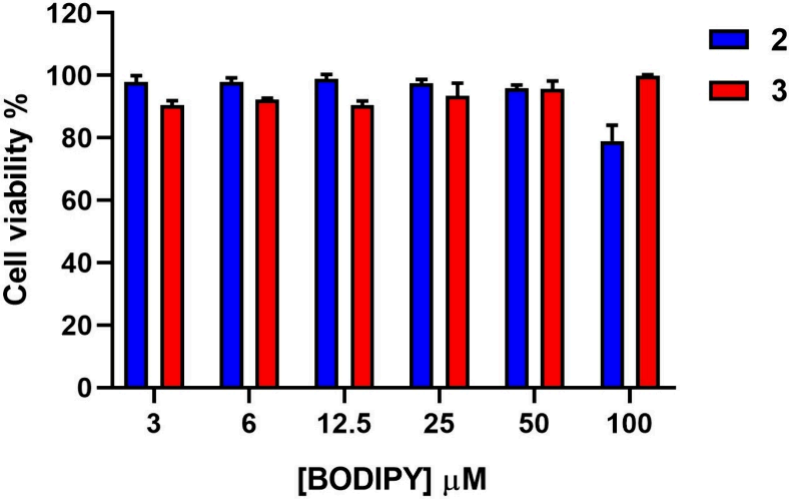
Viability assay in Hela cells. Cell viability was determined through the Resazurin assay after incubation for 24 h with different concentrations of BODIPY derivatives **2** and **3**. Data are presented as mean ± SEM from triplicate samples of two independent experiments.

### 3.4 Subcellular Localization

To investigate the internalization and intracellular localization of BODIPYs **2** and **3** within HeLa cells, colocalization experiments by confocal microscopy were performed with commercial probes for lipid droplets (LipidSpot) and for lysosomes (LysoTracker Deep Red).

Figure 8 depicts the fluorescence images of BODIPY **2** (green channel, λ_ex_ = 488 nm) and the commercial probes LipidSpot (red channel, λ_ex_ = 633 nm) and Hoechst 33342 (blue channel, λ_ex_ = 405 nm) within HeLa cells (Figure 7a–d) and the posterior analysis of their spatial overlap (Figure 8e,f).

**Figure 8:**
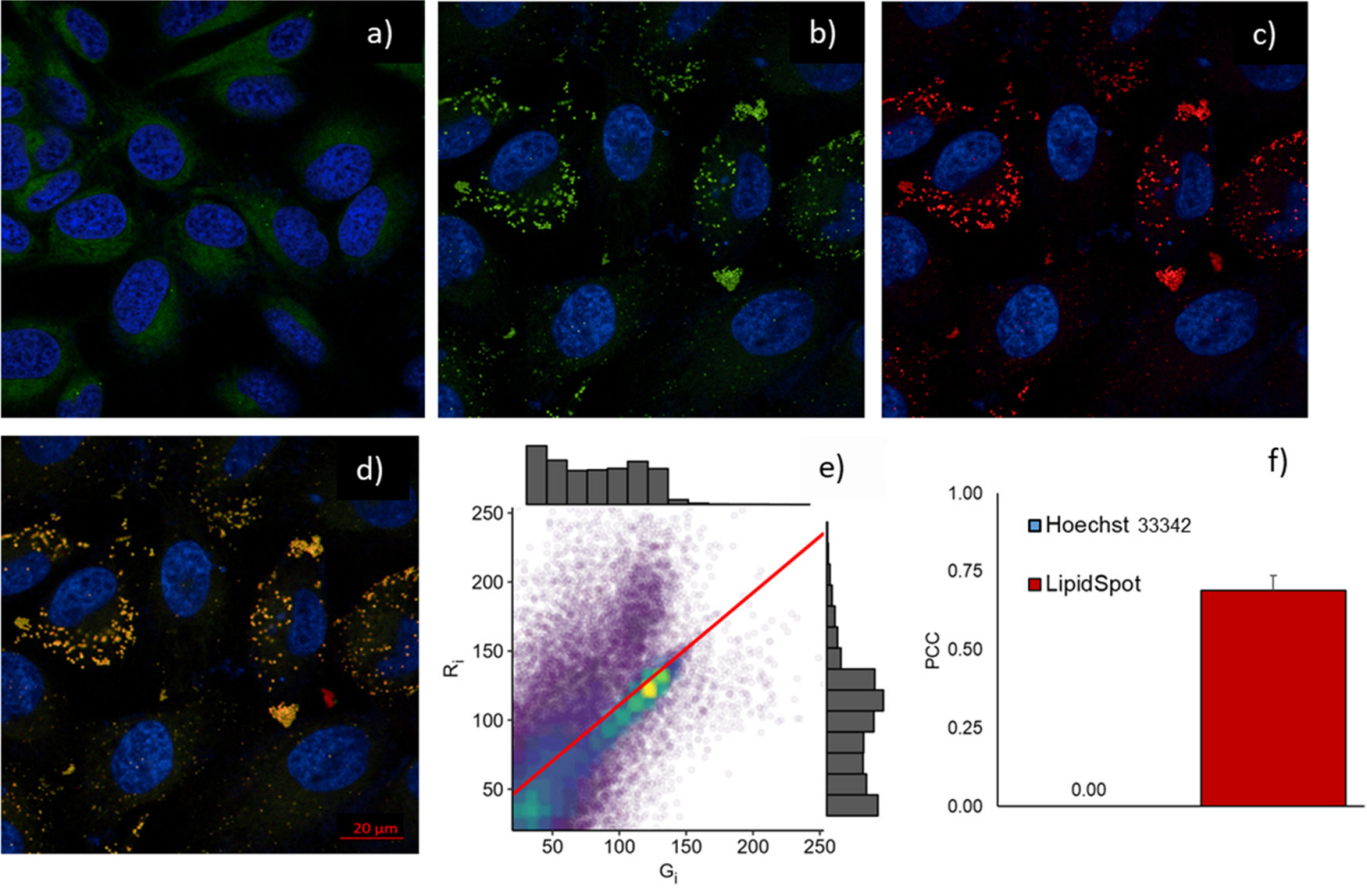
Confocal fluorescence images of HeLa cells treated with BODIPY **2** in the absence of oleic acid (a). Confocal fluorescence images of HeLa cells treated with BODIPY **2** (b); and LipidSpot (c) upon oleic acid treatment. Merge of Hoechst 33342 (blue channel, λ_ex_ = 405 nm), BODIPY **2** (green channel, λ_ex_ = 488 nm) and LipidSpot (red channel, λ_ex_ = 633 nm) (d). Scatterplot of the pixel intensity in the range of 20 to 255 for red (*R*_i_) and green (*G*_i_) channels (e). Pearson’s correlation coefficient (*r*) of BODIPY with LipidSpot and Hoechst 33342 dyes (f).

As shown in Figure 8a, BODIPY **2** was able to cross the cell membrane, yet the compound exhibited faint fluorescence intensity and nonspecific distribution within the cellular cytoplasm. Nevertheless, upon oleic acid treatment to induce the formation of lipid vesicles within the cells, the fluorescence pattern of BODIPY **2** suffers a clear change. In fact, it was observed a brighter fluorescence intensity and a specific pattern of the compound’s intracellular distribution (Figure 8b). Moreover, this shift in fluorescence distribution is consistent with the preferential localization of BODIPY **2** in lipid-rich environments, as observed in the computational studies conducted in *n*-octanol, which mimics the hydrophobic characteristics of lipid droplets. The computational results revealed a solvent-dependent shift in the absorption band of BODIPY **2**, suggesting that its photophysical properties are sensitive to the chemical environment.

Additionally, the merging of the fluorescence signal of compound **2** (green channel) and the commercial probe LipidSpot (red channel) resulted in a yellow pattern, indicating an extensive overlap (Figure 8d). These results were represented graphically in a scatterplot to compare pixel intensities for the red channel (R_i_) and green channel (G_i_) (Figure 8e), which revealed a clear positive linear correlation between these two variables. To quantify the degree of colocalization, we further employed Pearson’s correlation coefficient (*r*), where a value of 1 signifies a perfect and linear intertwining of channel intensities. As shown in Figure 8f, the *r* value for BODIPY **2** and LipidSpot dye was determined to be 0.70 ± 0.05, which corroborates an eminent spatial correlation between these two probes. In contrast, the *r* value calculated for BODIPY and Hoechst 33342 was zero, indicating uncorrelated fluorescence signals between them.

To investigate the intracellular distribution of compound **3**, its colocalization with lysosomes was assessed using LysoTracker Deep Red, a commercial probe that selectively labels these organelles. Figure 9 depicts the fluorescence images of BODIPY **3** (green channel, λ_ex_ = 488 nm) and commercial probes LysoTracker Deep Red (red channel, λ_ex_ = 633 nm) and Hoechst 33342 (blue channel, λ_ex_ = 405 nm) within HeLa cells (Figure 9a–c) and the posterior analysis of their spatial overlap (Figure 9d and **e**). It was observed that the BODIPY **3** was capable of readily diffusing through the cellular membrane, revealing an intense fluorescence signal in the intracellular environment (Figure 9a). For the colocalization between the BODIPY **3** and the Lysotracker, we combined the green channel with the red channel, and it was observed that the merged image (Figure 9c) resulted in a mainly yellow pattern, which is an indicator of overlap between the two dyes. Additionally, the green and the red color intensities were compared through one-to-one pixel matching and represented graphically in a scatterplot (Figure 9d), as a qualitative indicator of the colocalization degree between BODIPY **3** and LysoTracker, and the linear relationship was further studied through Pearson’s correlation coefficient (*r*) (Figure 9e). It was found that a value of 0.49 ± 0.10 between the green and the red channels reflected a moderate colocalization relationship between BODIPY **3** and LysoTracker, whereas an *r* value of −0.005 ± 0.113 between the green and the blue channels indicated uncorrelation between the intracellular localization of the BODIPY and the cell nucleus. This observation aligns with computational predictions, as the acidic lysosomal environment likely induces protonation of the benzimidazole and/or dimethylaminonaphthyl groups, thereby influencing the spectral properties within acidic organelles in live cells.

**Figure 9:**
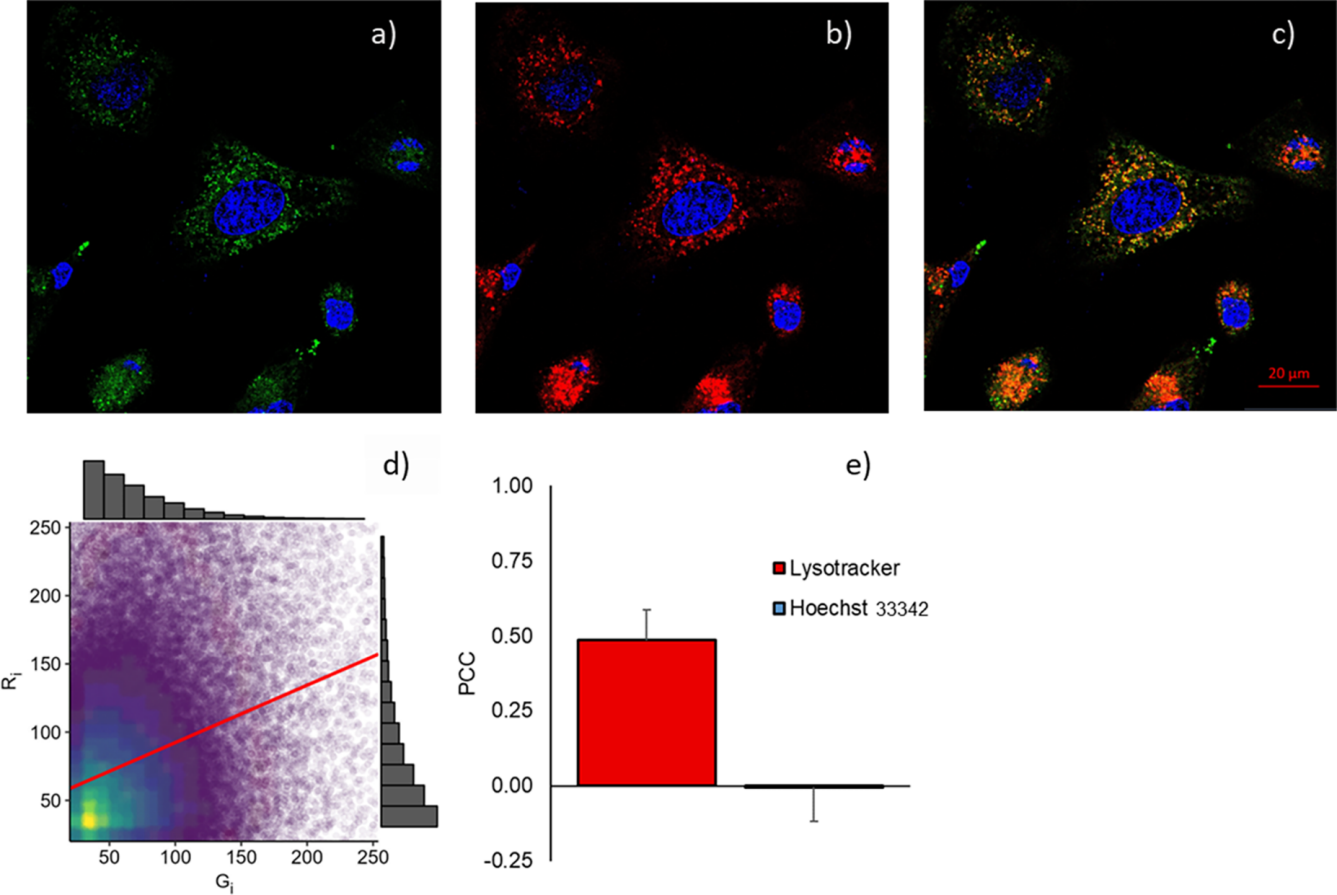
Confocal fluorescence images of HeLa cells treated with BODIPY **3** (a); and Lysotracker deep red (b). Merge of the Hoechst 33342 (blue channel, λ_ex_ = 405 nm), BODIPY **3** (green channel, λ_ex_ = 488 nm), and Lysotracker Deep Red (red channel, λ_ex_ = 633 nm) fluorescence signal (c). Scatterplot of the pixel intensity in the range of 20 to 255 for red (*R*_i_) and green (*G*_i_) channels (d). Pearson’s Correlation Coefficient (*r*) of BODIPY with Lysotracker and Hoechst dye (e).

Overall, these results indicate that BODIPY derivatives **2** and **3** are internalized by the cells. It was further demonstrated that BODIPY **2** has a pronounced affinity toward lipid clusters, while the fluorescence emission of BODIPY **3** is activated by the lower pH characteristic of lysosomes. Therefore, these compounds could be effectively employed as fluorescent markers to label these cellular structures within the cells.

### 3.5 Intracellular pH Assay

Computational studies and fluorescence spectra provided critical insights into the protonation behavior of compound **3** in different environments. The absorption spectra of compound **3** display significant pH-dependent shifts, suggesting heightened sensitivity of the photophysical properties of compound **3** to acidic environments. For further insight into the pH-dependent emission behavior of the BODIPY derivative **3** regarding the biological systems, HeLa cells were pretreated with buffer solutions at pH = 4.5 and 7 in the presence of nigericin, a known H^+^/K^+^ ionophore used to equilibrate the intracellular pH with the extracellular pH.^53^

The fluorescence intensity was plotted as the overall pixel intensity per cell for the green, red, and blue channels under each pH condition, along with the corresponding fluorescence gain (%) relative to pH = 7. It was observed that the intracellular fluorescence of BODIPY **3** was detected in both green (λ_ex_ = 488 nm) and red (λ_ex_ = 561 nm) channels. However, as shown in Figure 10a,d,g, the fluorescence intensity of BODIPY **3** within cells, when excited at 488 nm, exhibited a considerable increase when the intracellular pH decreased from 7 to 4.5, resulting in a fluorescence gain of 192.5%. Moreover, it should be noted that, while the emission of the BODIPY **3** was detected when excited at 561 nm, its intensity was no longer significantly influenced by the pH, with a fluorescence gain of only 34.8%, as depicted in Figure 10b,e,g. We propose that the pH sensitivity arises from the protonation–deprotonation equilibrium of the nitrogen atom from the benzimidazole moiety. The enhanced fluorescence of BODIPY **3** observed at lower pH levels can be attributed to the protonation of the benzimidazole moiety. This is consistent with previous DFT studies on 2-benzimidazole BODIPY derivatives, which demonstrated that photoinduced electron transfer (PET) occurs between the benzimidazole moiety and the BODIPY chromophore in its neutral form, leading to fluorescence quenching. In contrast, protonation of the benzimidazole moiety inhibits PET, thereby enhancing fluorescence emission.^24,30^ Furthermore, in agreement with the fluorescence spectra, the emission detected in the red channel could be attributed to the CT state. At pH = 7, the CT state is more populated, leading to an observable CT emission. At pH = 4.5, while some CT emission persists, it is less populated, and locally excited (LE) emission in the green channel becomes more prominent.

**Figure 10:**
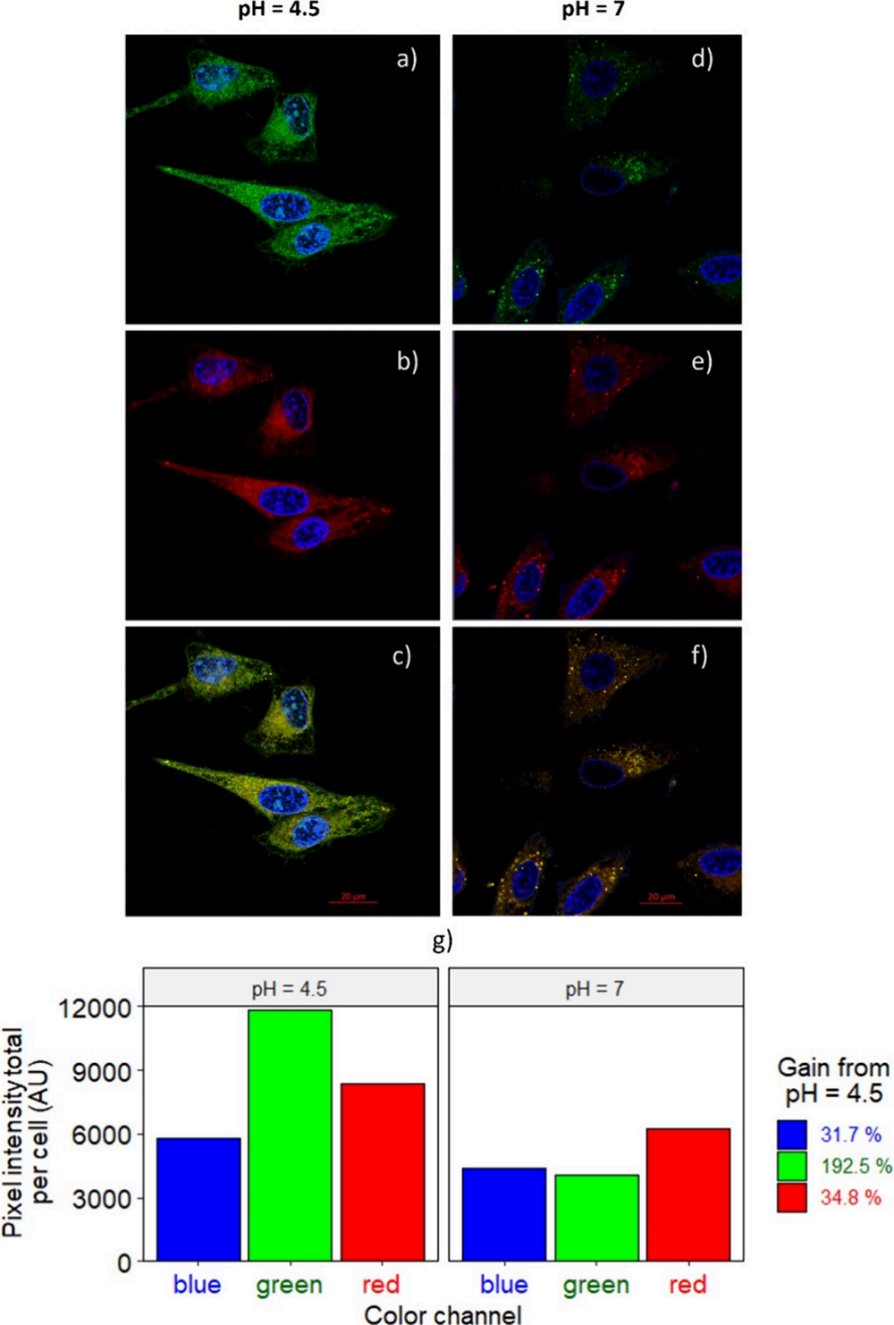
Confocal fluorescence images of HeLa cells incubated with the BODIPY derivative **3**. The cells were pretreated with buffer at pH = 4.5 (a–c); and pH = 7 (d–f) in the presence of nigericin. Fluorescence signal of Hoechst 33342 on the blue channel (λ_ex_= 405 nm, λ_em_ = 447 nm) and BODIPY **3** in the green channel (λ_ex_ = 488 nm, λ_em_ = 530 nm) and red channel (λ_ex_ = 561 nm, λ_em_ = 610 nm). Merged of blue, green, and red channels (c, f). Total pixel intensity per cell in the blue, green, and red channels at pH = 4.5 and pH = 7 with the fluorescence gain (%) from pH = 4.5, respectively (g).

Additionally, the fluorescence intensity of the Hoechst 33342 dye (blue channel) remained relatively unchanged across the two different pH conditions, as anticipated. This is consistent with the expected behavior of this commercial dye, considering that its emission intensity, when bound to nucleic acids, is not dependent on the intracellular pH.^61^ Thus, these results suggested that BODIPY derivative **3** may be employed as a fluorescence probe for imaging variations of intracellular pH in living cells.

## 4 Conclusions

The present study reports the influence of the chemical environment on the absorption spectra of *meso*-*N,N*-dimethylaminonaphthyl-BODIPY derivatives **2** and **3**, functionalized with formyl and benzimidazole electron-withdrawing groups at the 2-position, and their potential application as bioimaging probes.

The computational analysis of their absorption spectra revealed the limitations of traditional TDDFT approaches, which systematically failed to predict the experimentally observed absorption bands. In contrast, the MD/sTDA methodology provided significantly improved predictions, accurately capturing solvent-dependent spectral shifts and characteristic absorption bands of the BODIPY derivatives. The *in silico* studies effectively demonstrated that compound **2** displays distinct redshifts or blueshifts depending on the solvent, whereas BODIPY **3** exhibited more complex spectral properties, particularly in *n*-octanol, where a less polar environment may lead to more intense fluorescence signals.

Furthermore, *in vitro* studies demonstrated that compounds **2** and **3** exhibited good biocompatibility and could effectively penetrate the cell membrane. The subcellular localization studies demonstrated that BODIPY derivative **2** shows a strong affinity for lipid droplets, while BODIPY derivative **3** tends to accumulate in lysosomes. The intracellular pH assay confirms that BODIPY **3** can be employed as a fluorescence probe for the imaging of intracellular pH in living cells. Overall, these findings suggest that BODIPY derivatives **2** and **3** hold significant potential as fluorescent probes for tracking intracellular pH and labeling lysosomes and lipid droplets, offering potential for further insights into the dynamics of these organelles within living cells.

## Supplementary Material



## Notes

Characterization of BODIPY derivatives **1**–**3**; computational estimation of p*K*_a_; (Figure S1) fitting curve of the enthalpy difference between the neutral and conjugated acid forms of 13 reference compounds containing basic nitrogen atoms in amino-naphthyl or benzimidazole moieties; (Table S1) absolute enthalpies (in Hartrees), enthalpy difference (in kJ/mol) between the neutral and conjugated acid forms of 13 reference compounds containing basic nitrogen atoms in amino-naphthyl or benzimidazole moieties, and their respective p*K*_a_ used in the fitting procedure; (Table S2) enthalpy difference (in kJ/mol) between the neutral and conjugated acid forms of the BODIPY derivatives **1**, **2**, and **3**, and their estimated p*K*_a_ values, using the linear regression depicted in Figure S1; for compound **3**, p*K*_a__1_ was estimated by the difference between the pseudo-p*K*_a_ for process acid (DP) → acid (B) and p*K*_a__1_; (Figure S2) speciation of compound **3** with respect to the pH, using the estimated p*K*_a_ values calculated in this work; (Figure S3) experimental pH-dependent fluorescence response of BODIPY **3** within the 4–8.1 range (PDF)
