# Supplementary material for: meso-Dimethylaminonaphthyl-BODIPY Derivatives as Bioimaging Probes to Monitor Intracellular pH and Label Lysosomes and Lipid Droplets
Source: J Phys Chem B. 2025 Jul 1;129(28):7045–58. doi: 10.1021/acs.jpcb.5c00926 (PMC12818746; doi:10.1021/acs.jpcb.5c00926)
Supplement: Supplementary file 1 [file jp5c00926_si_001.pdf]

Supporting Information

meso-Dimethylaminonaphthyl-BODIPY Derivatives as Bioimaging Probes to Monitor Intracellular pH and Label Lysosomes and Lipid Droplets

Raquel C. R. Gonçalves,[1,2]* Sónia C. S. Pinto,[1]* Filipe Teixeira,[1] Efres Belmonte-Reche,[2] Milene Costa da Silva,[2] Juan Gallo,[2] Susana P. G. Costa,[1] M. Manuela M. Raposo[1]**

[1] Chemistry Centre of the University of Minho, Campus de Gualtar, 4710-057 Braga, Portugal.

[2] Advanced (magnetic) Theranostic Nanostructures Lab, International Iberian Nanotechnology Laboratory, Av. Mestre José Veiga s/n 4715-330 Braga, Portugal.

*Co-first authors; ** Corresponding author: mfox@quimica.uminho.pt

1. Characterization of BODIPYs 1-3......S2

*1.1. BODIPY derivative **1*** S2

*1.2. BODIPY derivative **2*** S2

*1.3. BODIPY derivative **3*** S3

2. Computational Estimation of pKa......S3

Figure S1. Fitting curve of the enthalpy difference between the neutral and conjugated acid forms of 13 reference compounds containing basic nitrogen atoms in amino-naphthyl or benzimidazole moieties. S4

Table S1. Absolute enthalpies (in Hartrees), enthalpy difference (in kJ/mol) between the neutral and conjugated acid forms of 13 reference compounds containing basic nitrogen atoms in amino-naphthyl or benzimidazole moieties, and their respective pKa used in the fitting procedure......S5

Table S2. Enthalpy difference (in kJ/mol) between the neutral and conjugated acid forms of the BODIPY derivatives 1, 2 and 3, and their estimated pKa values, using the linear regression depicted in **Figure S1**. For compound **3**, pKa_1 was estimated by the difference between the pseudo-pKa for process Acid (DP) → Acid (B) and pKa_1. ......S6

Figure S2. Speciation of compound **3** with respect to the pH, using the estimated pKa values calculated in this work. S6

Figure S3. Experimental pH-dependent fluorescence response of BODIPY **3** within the 4-8.1 range......S7

References......S7

1. Characterization of BODIPYs 1-3

The synthetic methods and partial characterization of compounds **1-3** can be found in references.[1,2] Mass spectrometry analysis was performed at the "C.A.C.T.I.—Unidad de Espectrometria de Masas" at the University of Vigo, Spain. Infrared spectra were measured on a Spectrum Two FT-IR Spectrometer in ATR mode – PerkinElmer. Here we report the FTIR analysis for all the compounds and HRMS analysis for compound **3**.

1.1. BODIPY derivative 1

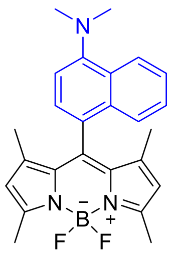

1

FTIR (solid): *ν* = 3585, 3164, 2953, 2836, 2786, 1539, 1502, 1457, 1396, 1372, 1302, 1184, 1152, 1035, 973, 837, 773, 616, 581 cm^{-1}

1.2. BODIPY derivative 2

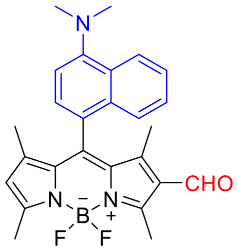

2

FTIR (solid): *ν* = 2922, 2834, 2782, **1662** (C=O), 1542, 1503, 1307, 1168, 1051, 981, 772, 575 cm^{-1}.

1.3. BODIPY derivative 3

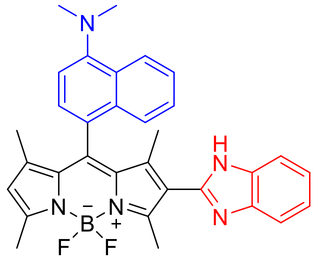

3

FTIR (solid): ν = 3073, 2927, 2845, 2781, 1540, 1435, 1401, 1314, 1182, 984, 743, 615, 548 cm^{-1}.

MS (ESI) *m/z* (%): 535 ($[M + 2]^{+\bullet}$, 30), 534 ($[M + 1]^{+\bullet}$, 80), 533 ($[M]^{+\bullet}$, 21), 267 (100); HRMS (ESI) *m/z*: $[M + 1]^{+\bullet}$ calcd for $C_{32}H_{31}BF_2N_5$, 534.2635; found 534.2633.

2. Computational Estimation of pKa

The PubChem database[3] was scanned for compounds with known pKa associated to the nitrogen atom of either benzimidazole or amino-naphthyl groups. The geometry of each of these compounds, and their respective conjugated acids, was optimized using DFT at the ωB97X-D3BJ/Def2-TZVP level of theory, and their zero-point vibrational energy, absolute enthalpies and Gibbs energies were calculated via the harmonic oscillator approximation, as detailed in the Main Text. Considering the process associated with pKa,

$$AH^+ \,(aq) + H_2O \,(l) \rightleftharpoons A \,(aq) + H_3O^+ \,(aq)$$

we postulated that the contributions of water and the oxonium cation to the energy balance would be constant along the series of compounds, hence, we sought to approximate pKa as a linear function of the energy difference between AH^+ and A, in aqueous media (modelled using the C-PCM model). The exploration of four possible thermochemistry parameters (electronic energy, electronic energy with zero-point energy correction, enthalpy and Gibbs energy) shown that the difference in enthalpy between AH^+ and A yielded the best linear fitting to the known values of pKa, shown in **Figure S1**, using the data provided in **Table S1**. The fitting equation

$$pKa = 0.05635\ (\Delta H/\ kJ.mol^{-1}) - 60.18$$

was then applied to the enthalpy balance concerning compounds **1**, **2**, and **3**, and their respective conjugated acids. The estimated values of pKa for compounds **1** and **2**, as well as pKa_2 of compound **3** were estimated directly from the linear regression data, shown in **Table S2**. The approach described here approximates pKa to a state function, hence, the value of pKa1 of compound **3** was derived by subtraction of the pseudo-pKa value found for the deprotonation of the DP form of compound **3** resulting in its immediate conjugated acid:

$$AH_2^{2+}\,(aq) + H_2O\,(l) \rightleftharpoons AH^{+}\,(aq) + H_3O^{+}\,(aq)$$

and its pKa_2 (considering protonation/deprotonation of the benzimidazole moiety).

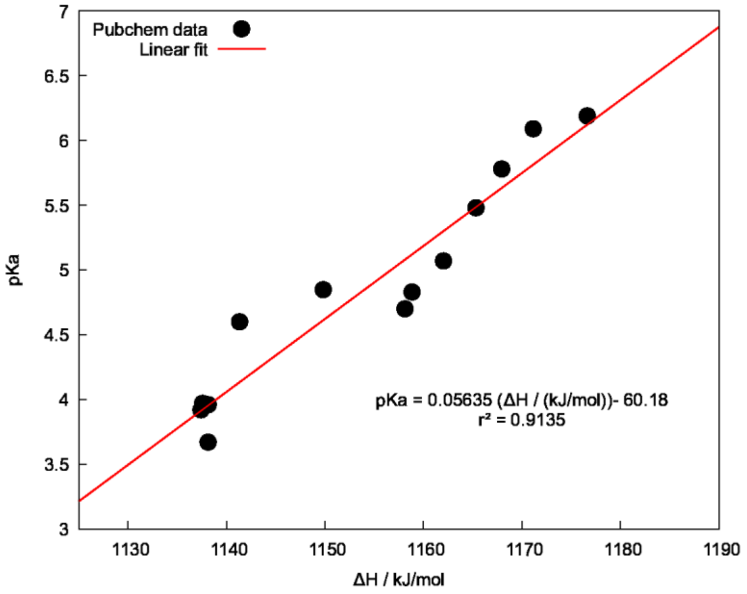

Figure S1. Fitting curve of the enthalpy difference between the neutral and conjugated acid forms of 13 reference compounds containing basic nitrogen atoms in amino-naphthyl or benzimidazole moieties.

Table S1. Absolute enthalpies (in Hartrees), enthalpy difference (in kJ/mol) between the neutral and conjugated acid forms of 13 reference compounds containing basic nitrogen atoms in amino-naphthyl or benzimidazole moieties, and their respective pKa used in the fitting procedure.

Compound	Speciation	H / Eh	ΔH / kJ.mol^{-1}	pKa
1-Methylimidazole	Acid	-266.02434		
1-Methylimidazole	Neutral	-265.64355	999.76	6.95
1-Naphthylamine	Acid	-441.87904		
1-Naphthylamine	Neutral	-441.44582	1137.41	3.92
2-Methylbenzimidazole	Acid	-419.83352		
2-Methylbenzimidazole	Neutral	-419.38536	1176.63	6.19
5,6-Dimethylbenzimidazole	Acid	-459.14863		
5,6-Dimethylbenzimidazole	Neutral	-458.70256	1171.14	6.09
5-Amino-1-naphthol	Acid	-517.14520		
5-Amino-1-naphthol	Neutral	-516.71171	1138.14	3.96
5-Amino-2-naphthol	Acid	-517.14577		
5-Amino-2-naphthol	Neutral	-516.71249	1137.57	3.97
5-Chloro-1*H*-benzimidazole	Acid	-840.11961		
5-Chloro-1*H*-benzimidazole	Neutral	-839.67851	1158.12	4.7
5-Methylbenzimidazole	Acid	-419.82350		
5-Methylbenzimidazole	Neutral	-419.37865	1167.94	5.78
Aniline	Acid	-288.16248		
Aniline	Neutral	-287.72778	1141.33	4.6
Benzimidazole	Acid	-380.49675		
Benzimidazole	Neutral	-380.05290	1165.33	5.48
N-Methylnaphthalen-1-amine	Acid	-481.18988		
N-Methylnaphthalen-1-amine	Neutral	-480.75639	1138.13	3.67
N-Methylaniline	Acid	-327.47595		
N-Methylaniline	Neutral	-327.03801	1149.82	4.85
N,N-Dimethyl-1-naphthylamine	Acid	-520.50515		
N,N-Dimethyl-1-naphthylamine	Neutral	-520.06377	1158.84	4.83
N,N-Dimethylaniline	Acid	-366.79048		
N,N-Dimethylaniline	Neutral	-366.34788	1162.03	5.07

Table S2. Enthalpy difference (in kJ/mol) between the neutral and conjugated acid forms of the BODIPY derivatives 1, 2 and 3, and their estimated pKa values, using the linear regression depicted in **Figure S1**. For compound **3**, pKa_1 was estimated by the difference between the pseudo-pKa for process Acid (DP) → Acid (B) and pKa_1.

Compound	Process	ΔH / $kJ.mol^{-1}$	Predicted pKa
01	Acid → Neutral	1130.47	3.51
02	Acid → Neutral	1129.72	3.47
03	Acid (A) → Neutral	1129.27	3.44
03	Acid (B) → Neutral	1172.99	5.91 (pKa_2)
03	Acid (DP) → Acid (A)	1172.66	5.89
03	Acid (DP) → Acid (B)	1128.94	3.42
03	Acid (DP) → Neutral	2301.93	2.44 (pKa_1)

The values of pKa for compound **3** were then used as parameters in a multiple-equilibrium problem between four species (neutral, acid (A), acid (B) and DP) at a given pH, using the Solver functionality of OpenOffice's Calc spreadsheet, which yielded the speciation curves depicted in **Figure S2**, used to estimate the speciation of compound **3** at pH 4.

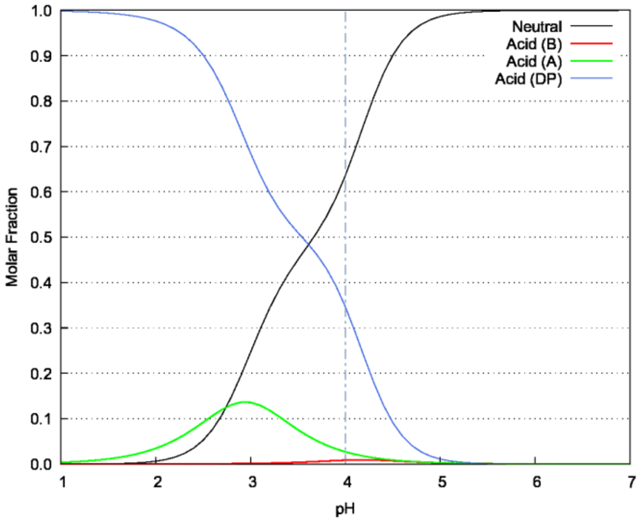

Figure S2. Speciation of compound **3** with respect to the pH, using the estimated pKa values calculated in this work.

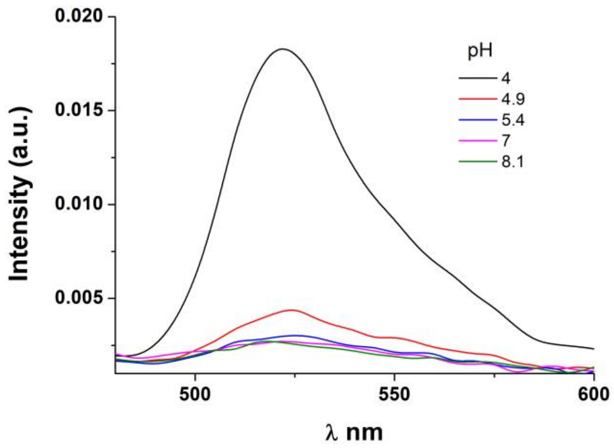

Figure S3. Experimental pH-dependent fluorescence response of BODIPY **3** within the 4-8.1 range.

References:

(1) Gonçalves, R. C. R.; Nogueira, M. B.; Costa, S. P. G.; Raposo, M. M. M. BODIPY Derivatives: Synthesis and Evaluation of Their Optical Properties. *Proceedings* **2019**, *9*, 10. https://doi.org/10.3390/ecsoc-22-05700.

(2) Gonçalves, R. C. R.; Pina, J.; Costa, S. P. G.; Raposo, M. M. M. Synthesis and Characterization of Aryl-Substituted BODIPY Dyes Displaying Distinct Solvatochromic Singlet Oxygen Photosensitization Efficiencies. *Dyes Pigm.* **2021**, *196*, 109784. https://doi.org/10.1016/j.dyepig.2021.109784.

(3) Kim, S.; Chen, J.; Cheng, T.; Gindulyte, A.; He, J.; He, S.; Li, Q.; Shoemaker, B. A.; Thiessen, P. A.; Yu, B.; Zaslavsky, L.; Zhang, J.; Bolton, E. E. PubChem 2025 update. *Nucleic Acids Res.* **2025**, *53*(D1), D1516–D1525. https://doi.org/10.1093/nar/gkae1059.
